# Proliferation Index: A Continuous Model to Predict Prognosis in Patients with Tumours of the Ewing's Sarcoma Family

**DOI:** 10.1371/journal.pone.0104106

**Published:** 2014-08-26

**Authors:** Samantha Brownhill, Dena Cohen, Sue Burchill

**Affiliations:** 1 Children's Cancer Research Group, Leeds Institute of Cancer and Pathology, St James's University Hospital, Leeds, United Kingdom; 2 Clinical Trials Research Unit, Leeds Institute of Cancer and Pathology, St James's University Hospital, Leeds, United Kingdom; Johns Hopkins University, United States of America

## Abstract

The prognostic value of proliferation index (PI) and apoptotic index (AI), caspase-8, -9 and -10 expression have been investigated in primary Ewing's sarcoma family of tumours (ESFT). Proliferating cells, detected by immunohistochemistry for Ki-67, were identified in 91% (91/100) of tumours with a median PI of 14 (range 0–87). Apoptotic cells, identified using the TUNEL assay, were detected in 96% (76/79) of ESFT; the median AI was 3 (range 0–33). Caspase-8 protein expression was negative (0) in 14% (11/79), low (1) in 33% (26/79), medium (2) in 38% (30/79) and high (3) in 15% (12/79) of tumours, caspase-9 expression was low (1) in 66% (39/59) and high (3) in 34% (20/59), and caspase-10 protein was low (1) in 37% (23/62) and negative (0) in 63% (39/62) of primary ESFT. There was no apparent relationship between caspase-8, -9 and -10 expression, PI and AI.

PI was predictive of relapse-free survival (RFS; p = 0.011) and overall survival (OS; p = <0.001) in a continuous model, whereas AI did not predict outcome. Patients with tumours expressing low levels of caspase-9 protein had a trend towards a worse RFS than patients with tumours expressing higher levels of caspase-9 protein (p = 0.054, log rank test), although expression of caspases-8, -9 and/or -10 did not significantly predict RFS or OS. In a multivariate analysis model that included tumour site, tumour volume, the presence of metastatic disease at diagnosis, PI and AI, PI independently predicts OS (p = 0.003). Consistent with previous publications, patients with pelvic tumours had a significantly worse OS than patients with tumours at other sites (p = 0.028); patients with a pelvic tumour and a PI≥20 had a 6 fold-increased risk of death. These studies advocate the evaluation of PI in a risk model of outcome for patients with ESFT.

## Introduction

Ewing's sarcoma family of tumours (ESFT) can arise in bone or soft tissue sites at any age, but most frequently are diagnosed in bony sites in children and young adults [1]. Five year survival rates for patients diagnosed with localised disease are between 60 and 70%, although outcome for patients with metastasis is lower despite multimodality treatment incorporating combination chemotherapy, surgery and radiotherapy. Since improved patient outcome is anticipated by adapting therapy based on risk, a number of prognostic clinical factors have been described including the presence of metastatic disease at the time of diagnosis [2], tumour volume (greater than 200 ml) [3] and pelvic primary tumours [4], [5] at diagnosis in patients with localised disease. Despite these observations there is currently no universally accepted informative staging system in ESFT.

High proliferation index (PI), is reported to predict poor outcome in colon carcinoma [6], renal cell carcinoma [7], cervical carcinoma [8], neuroblastoma [9], bladder [10] and breast [11]–[13] cancer, as well as ESFT [14]–[16]. Although apoptotic index (AI) affects tumour viability and growth, the relationship between AI and prognosis is controversial and has not previously been investigated in ESFT. High AI is predictive of increased survival in patients with osteosarcoma [17] and gastric cancer [18], and low AI has been associated with high grade tumours of the ovary [19], kidney [20] and colon [6]. In contrast, low AI has been associated with a higher mean survival in childhood acute lymphoblastic leukaemia (ALL) [21] and patients with colorectal carcinoma [22].

Since tumour growth reflects the number of both proliferating and apoptotic cells, we have hypothesised that PI and AI may more reliably predict outcome than PI alone. This hypothesis is supported by studies in patients with adenocarcinoma of the uterine cervix, where the ratio of PI/AI was predictive of survival but not PI or AI alone [23]. The relationship between expression of the effector caspases, PI, AI and outcome is poorly investigated, caspase-8 being the most frequently studied. Methylation of the *CASP8* gene has been associated with low levels of caspase-8 expression, reduced response to chemotherapeutic agents and poor outcome in a number of different cancer types including medulloblastoma [24] and neuroblastoma [25], [26], although this remains controversial at least in neuroblastoma [27]. Lack or low caspase-8 expression has also been reported in a number of other cancer types including osteosarcoma [28] and squamous cell carcinoma of the tongue [29]. In ESFT cell lines [30] and tumours [31], expression is variable [32], [33] and is reported to have no correlation with outcome [33].

Although caspase-9 and -10 are executors of apoptosis, little is known of their prognostic value in primary tumours. Expression of cleaved caspase-9 protein correlates with a longer OS in patients with Hodgkin's lymphoma [34], although the clinical significance of low caspase-9 expression in colon carcinoma [35], medulloblastoma [24] and gastric carcinoma [36] remains unclear. Expression of caspase-10 is also low in gastric carcinoma [36], rectal [37] and colorectal [38] cancers. The aims of this study were to examine PI, AI and expression of caspases-8, -9 and -10 in a panel of primary ESFT to evaluate and compare their prognostic power.

## Materials and Methods

### Clinical samples

Tumour samples were collected at diagnosis from 105 patients with ESFT, diagnosis was confirmed by reverse transcriptase polymerase chain reaction (RT-PCR) for the *EWS-ETS* gene rearrangements in 80/105 tumours. Of the remaining 25 tumour samples, CD99 expression was confirmed by immunohistochemistry (IHC) for MIC-2 in 25/25 tumours. It was not possible to analyse all tumours for all markers due to limited amounts of material; suitable material was available to analyse PI in 100/105, AI in 79/105, caspase-8 in 79/105, caspase-9 in 59/105 and caspase-10 in 62/105 of cases. The presence of metastases was detected by conventional imaging and examination of bone marrow aspirates by light microscopy.

### Ethics statement

Ethical approval for this study was obtained from Trent Multi-centre Research Ethics Committee (MREC 98/4/023; MREC 98/0/44). Informed written consent was obtained from all participants.

### Immunohistochemistry for Ki-67, caspases-8, -9 and -10

Immunohistochemistry and antigen retrieval were optimised using sections (5 µm) of frozen or formalin fixed-paraffin embedded (FF-PE) ESFT cell pellets; sections of TC-32 cell pellets were included as positive controls in subsequent assays. Sequential sections (5 µm) of frozen (n = 86) and FF-PE (n = 19) primary ESFT were analysed. FF-PE sections were hydrated and antigen retrieval was performed by boiling the sections in citric acid buffer for 10 minutes prior to analysis. Sections processed without primary antibody or with isotype matched antibody were included as negative controls. All sections were counterstained with haematoxylin and mounted in DePeX mounting medium.

Proliferating cells were detected by immunohistochemistry for Ki-67 using a mouse monoclonal antibody (1∶100; Dako, Cambridgeshire, UK) and 3 stage peroxidase [39]. PI = [number of Ki-67 positive cells ÷ number of cells scored]×100; 100 cells were scored in 3 different fields of view by two independent observers.

Caspase-8 and caspase-9 proteins were detected using the Rabbit Envision Kit (Dako) and caspase-8 (1 in 50; Santa Cruz, Heidelberg, Germany) or caspase-9 (1 in 100; Abcam, Cambridge, UK) antibodies. Prior to incubation with primary antibody overnight at 4°C, sections were incubated with normal goat serum (1 in 10; Dako) for 1 hour at room temperature, wash steps were performed with TBS plus 1% Tween 20. Caspase-10 protein was detected using caspase-10 primary antibody (1 in 150; Santa Cruz) and the Goat Vectastain ABC kit (Vector Laboratories, Peterborough, UK); sections were incubated with primary antibody for one hour at 37°C and antibody was visualised using DAB+ substrate (Dako).

### Identification of apoptotic cells by terminal deoxynucleotidyl transferase (TdT) mediated dUTP nick-end labelling (TUNEL)

Apoptotic cells were detected by TUNEL using the ApopTag Peroxidase *In Situ* Apoptosis Detection Kit (Millipore, UK), according to manufacturer's instructions. AI = [number of TUNEL positive cells ÷ number of cells scored]×100; 100 cells were scored in three different fields of view by two independent observers.

### Scoring of immunohistochemistry

Sections were visualised by light microscopy (Zeiss Axioplan microscope, Zeiss, UK) and scored using the following criteria. Caspase-8 expression was negative (0), low (1), medium (2) or high (3) based on intensity of staining compared to a caspase-8 positive (2, tumour 11) and negative (0, tumour 45) tissue. Caspase-9 expression was scored as negative (0), low (1), medium (2) or high (3) dependant on staining intensity compared to tissue with high (3, tumour 55) or low (1, tumour 57) expression, and caspase-10 as negative (0), low (1), medium (2) or high (3) compared to a caspase-10 expressing tumour (1, tumour 1) and a negative (0, tumour 57). Staining was independently evaluated by two observers.

### Statistical analysis

All statistical analyses were carried out using SAS v9.2 (SAS Institute Inc., Cary, NC, USA). RFS was calculated from the date of diagnosis to relapse or death, or the date of last assessment without event. OS was defined as the time from diagnosis to death or the date last seen alive. Univariate analyses explored whether a number of covariate factors predict prognosis: age group (<14, ≥14) [40]; tumour site (pelvic, other) [4]; tumour volume (<200 ml, ≥200 ml) [3]; metastases at diagnosis (any, none) [2]; response to treatment (<90%, ≥90%) [41]; PI (as a continuous variable); AI (as a continuous variable); caspases-8, -9 and -10 (0/1, 2/3). RFS and OS curves were calculated using the Kaplan-Meier method by each covariate group, and the results for each compared using the log rank test. Caution was taken in statistical interpretation to minimise the effect of multiplicity, and the p-values were compared to 0.01 in order to control the type 1 error rate.

Cox proportional hazards regression was carried out on OS, incorporating the most significant covariates to assess which are prognostic in a multivariate setting. A stepwise procedure was used, with significance level for entering an explanatory variable into the model set to 0.05 and significance level for removing an explanatory variable from the model set to 0.1.

An investigation was carried out into the optimal cut-point choice for PI. PI was investigated as a continuous covariate within a Cox model; the log-rank value was calculated over the whole range of cut-points to visualise any patterns, and the cut-point log rank values were compared to those when the data is described as a continuous covariate. Fractional polynomials were calculated to investigate how the data could best be described as a continuous covariate, using a series of predefined transformations of predictor variables [42]. If the cut-point describes the data significantly better than a fitted model, it is likely that the cut-point effect is real; otherwise the survival may be related to the PI on a continuous scale.

## Results

Frozen and FF-PE tumour distribution with age at diagnosis, tumour site and PI confirmed that frozen and FF-PE tumours could be considered as a single sample set (Table S1). Patient and tumour details, PI, AI and protein expression profiles are summarised in Table S2.

### Prognostic power of clinical parameters

**Figure 1:**
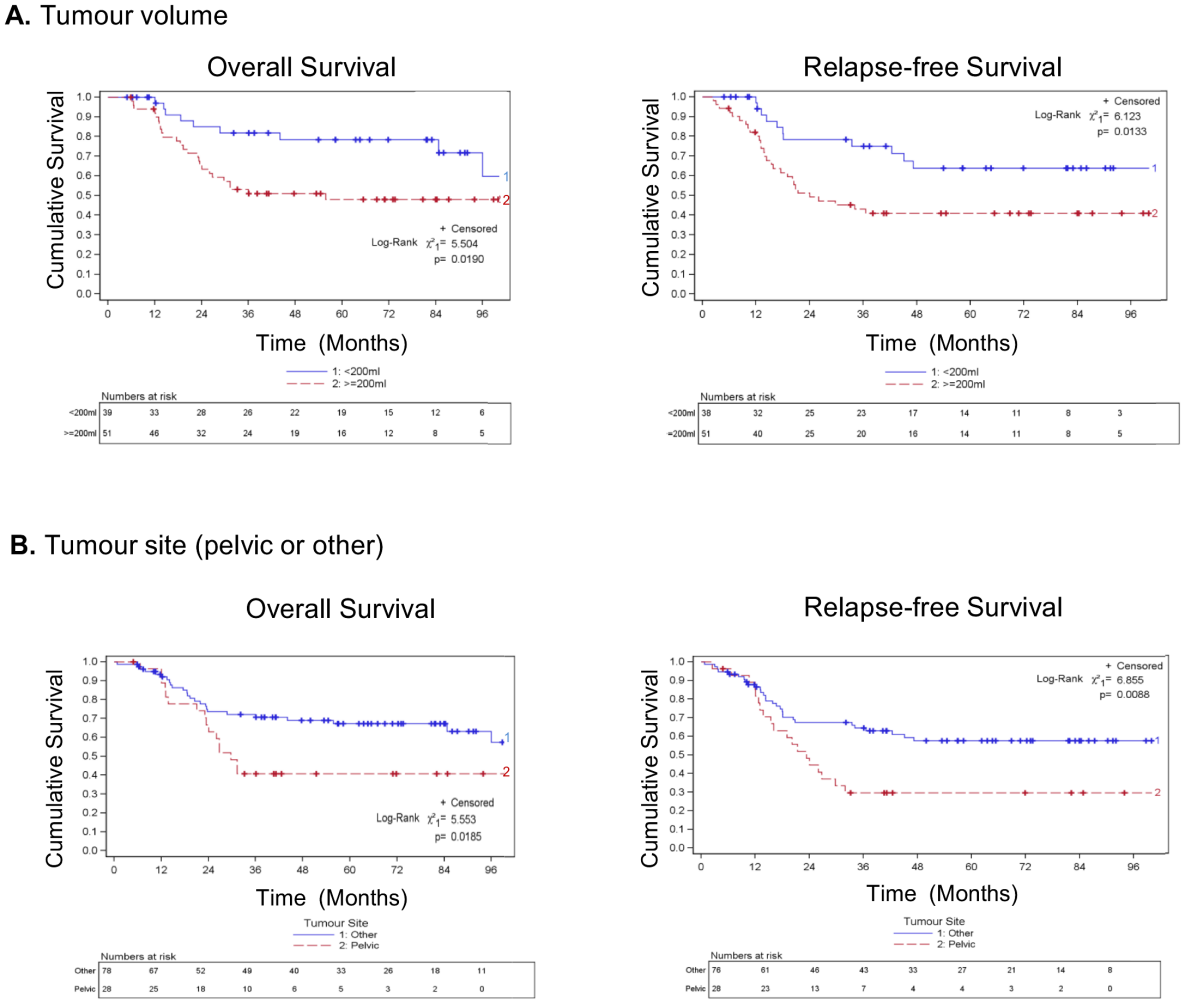
A. Kaplan Meier survival plots comparing the overall and relapse-free survival of patients with a tumour volume ≥200 ml with that of patients with a tumour volume <200 ml, p = 0.019 and 0.0133 respectively; log rank test. Crosses indicate censored events. B. Kaplan Meier survival plots to compare the overall and relapse-free survival of patients with pelvic tumours to that of patients with tumours at other sites, p = 0.0185 and 0.0088 respectively; log rank test. Crosses indicate censored events.

The prognostic power of parameters previously reported to be of clinical value was investigated in this patient cohort. Consistent with previous studies a tumour volume ≥200 ml was predictive of a poor OS and RFS (p = 0.019 and 0.013 respectively, log rank test, Figure 1A) compared to a tumour volume <200 ml. Furthermore, patients with pelvic tumours had a worse OS and RFS (p = 0.019 and 0.009 respectively, log rank test, Figure 1B) than patients with tumours presenting in other sites. Age of the patient (<14 years compared to ≥14 years) and response to treatment (<90% necrosis of tumour post-treatment compared to ≥90% necrosis) did not predict outcome in this cohort. The presence of metastatic disease at diagnosis detected by conventional imaging and examination of bone marrow aspirates by light microscopy for tumour cells did not predict outcome, although there was a non-significant trend towards patients with metastasis at diagnosis having a worse OS (p = 0.099, log rank test). Site of metastasis was available for 20/32 (63%) patients with metastasis; patients with bone metastasis at diagnosis had a significantly worse OS and RFS (p = <0.001 and <0.001 respectively, log rank test) than patients with no metastatic disease or metastasis at other sites.

### Proliferation and apoptosis in primary ESFT

**Figure 2:**
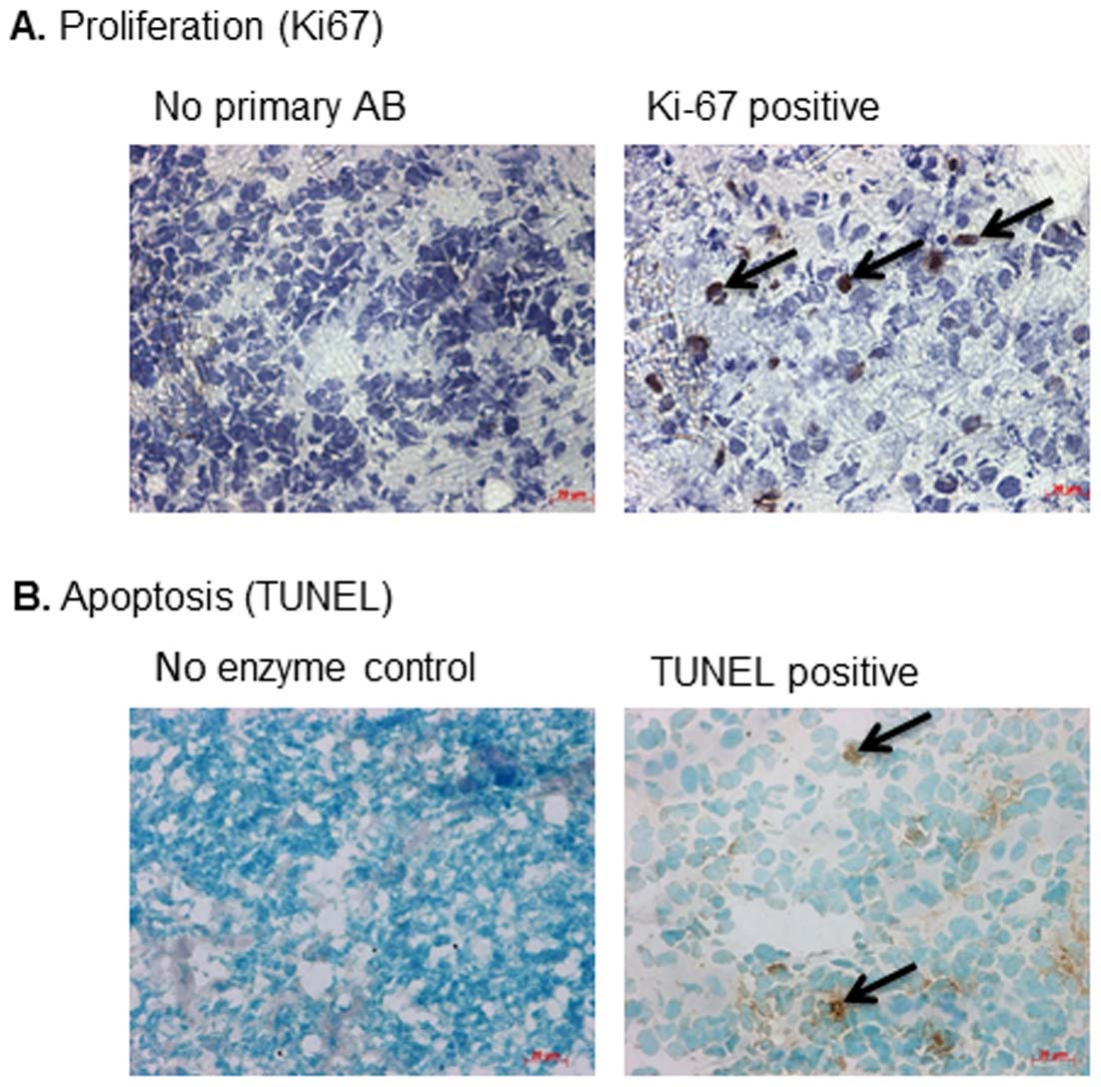
A. Immunohistochemistry for Ki-67 performed on primary ESFT (5 µm). Ki-67 protein was visualised with the DAB substrate, proliferating cells are identified by Ki-67 positive nuclei (see arrows). Immunohistochemistry performed in the absence of primary antibody was included to control for non-specific binding of the secondary antibody (No primary AB). Magnification ×400. B. TUNEL on primary ESFT (5 µm). Apoptotic cells were identified by TUNEL positive nuclei (see arrows). The TUNEL assay was performed in the absence TdT enzyme to control for non-specific staining (No enzyme). Magnification ×400.

Proliferating cells were identified in 91% (91/100) of tumours with a median PI of 14 (range = 0–87) (Figure 2A; Table S2). Fractional polynomial analysis showed that none of the transformations were significantly better than the simple linear model, suggesting that PI is linearly related to OS. The log-rank values over the entire range of cut-points show that there is no optimal cut-point, and since the log-rank values were not substantially higher than those for the linear model supports the conclusion that PI is a truly continuous variable.

A Cox regression for OS incorporating PI as a continuous covariate demonstrates that those patients with tumours that have a high PI at diagnosis will have a worse OS than those patients with a lower PI (χ^2^ = 11.5, p<0.001, HR = 1.04, 95% CI = 1.02–1.06). PI was also included in a multivariate analysis for OS along with the covariates tumour site, tumour volume, metastases at diagnosis and AI. In this analysis PI remained strongly significant (χ^2^ = 9.11, p = 0.003, HR = 1.04, 95% CI = 1.01–1.06), along with tumour site (χ^2^ = 4.85, p = 0.028, HR = 2.81, 95% CI = 1.12–7.04), where patients with pelvic tumours had a worse outcome. With these two variables in the model, none of the other factors significantly added to the prediction of OS. Patients with a pelvic tumour and a high PI have a worse OS than those with either factor alone; patients with a pelvic tumour and a PI≥20 have a HR of 5.8. A Cox regression for RFS incorporating PI as a continuous covariate demonstrates that those patients with tumours that have a high PI at diagnosis will have a worse RFS than those patients with a lower PI (χ^2^ = 6.5, p = 0.011, HR = 1.03, 95% CI = 1.01–1.06). Apoptotic cells were identified in 96% (76/79) of ESFT with a median AI of 3 (range = 0–33) (Figure 2B; Table S2); AI does not predict OS (p = 0.137, Cox regression) or RFS (p = 0.596, Cox regression).

### Caspase-8, -9 and -10 protein expression in primary ESFT

**Figure 3:**
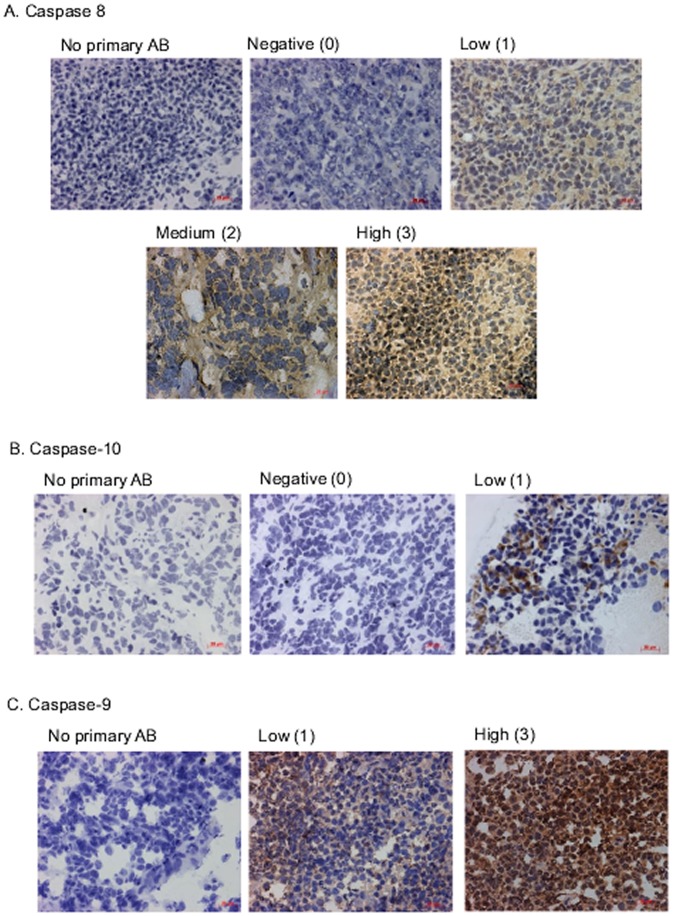
Immunohistochemistry on primary ESFT (5 µm) for caspases-8, -9 and -10. Immunohistochemistry in the absence of primary antibody was to control for non-specific binding of the secondary antibody (No primary AB). Magnification of images = ×400. Caspase expression was scored as negative (0), low (1), medium (2) or high (3). A. Caspase-8 protein expression. B. Caspase-10 protein expression. C. Caspase-9 protein expression.

Caspase-8 protein was heterogeneously expressed in the cytoplasm (Figure 3A; Table S2); negative (0) in 11/79 (14%), low (1) in 26/79 (33%), medium (2) in 30/79 (38%) and high (3) in 12/79 (15%) primary ESFT. Caspase-8 protein expression did not predict OS or RFS (p = 0.250 and 0.235 respectively, log rank test). Caspase-10 protein was expressed at a low level (1) in the cytoplasm of 23/62 (37%) of tumours and not expressed (0) in 39/62 (63%; Figure 3B; Table S2) and did not predict OS (p = 0.732, log rank test) or RFS (p = 0.693, log rank test).

Caspase-9 protein was detected in the cytoplasm and nucleus of ESFT (Figure 3C; Table S2), expression was low (1) in 39/59 (66%) and high (3) in 20/59 (34%) of tumours. Expression of caspase-9 was not predictive of OS (p = 0.275, log rank test), however, patients with tumours expressing low levels of caspase-9 protein had a strong trend towards a worse RFS than patients with tumours expressing high levels of caspase-9 protein (p = 0.054, log rank test, Figure S1). Nuclear or cytoplasmic expression did not appear to contribute to the prognostic value of low and high expression across the tumour. This is worthy of investigation in a validation tumour cohort.

## Discussion

In this study we demonstrate for the first time that high numbers of proliferating cells in primary ESFT are predictive of a worse RFS and OS in a continuous model. In a multivariate analysis, high PI was predictive of poor OS independent of tumour site, tumour volume, metastasis at diagnosis or AI. The prognostic value of PI is consistent with results from univariate [14], [16] and multivariate [15] discontinuous studies in ESFT. However there is considerable variation in the level above which a tumour is scored as having a high number of proliferating cells; in the published literature high PI has being defined as ≥50% [16] or >5% [15]. This wide discrepancy can be explained by the variable having a truly continuous distribution. One group has reported no association between PI and outcome using an arbitrary cut-point of 20 (based on the literature at the time, p = 0.71) or a statistically defined cut-point of 8.3 (generated by analysis of the ROC curve, p = 0.06) [14]; this most likely reflects the continuous relationship between PI and outcome and the distribution of patients in this small group (n = 37). We have detected proliferating cells in 91% of ESFT, higher than previous studies where they reported Ki-67 staining in 57% [14] and 34% [15]. This is likely to be due to the fact that these authors only reported Ki-67 expression once the PI had reached the cut points of 8.3% and 5% respectively, whereas we have reported tumours to be positive with a PI of 1% or greater. Since a higher PI is predictive of worse OS in a continuous model, patient risk should optimally be defined by incorporating the PI value rather than an arbitrary cut-point into a predictive model. Alternatively the introduction of PI as a biomarker of risk into the clinic may be enabled by defining a cut-point that identifies patients at ultra-high risk, for whom current treatment offers no benefit and might be offered novel investigational treatments. For example, we have identified that patients with a PI value of ≥20 (33% of patients in this study) have a two-fold increased risk of death (HR = 2.0) or relapse (HR = 1.9), whereas patients with a pelvic tumour and a PI≥20 have a 6 fold-increased risk of death (HR = 5.8) suggesting PI and tumour site should be included in a risk model for patients with ESFT. The quantification of PI by immunohistochemistry for Ki-67 protein is a robust reproducible assay that can be performed reliably on paraffin embedded tumour at low cost, and so may readily be introduced into pathology practice. The utility of this marker, and others where quantification is important, may benefit from an observer independent assessment using digital imaging to allow high-throughput analysis and to overcome subjectivity and heterogeneity of expression. A recent study found that Ki-67 quantification by digital image analysis and manual counting were highly concordant [43]. It is now important to evaluate the prognostic value of PI as a continuous variable with other prognostic biomarkers to identify the most clinically informative algorithm of risk for patients with ESFT.

The number of apoptotic cells was low in the majority of primary ESFT, and AI did not predict prognosis in the univariate or multivariate models. This is the first study to describe this relationship in ESFT. AI has been correlated with survival in other cancers with contradictory findings, however analysis has been based values above and below cut points rather than as a continuous variable [17]–[19]. An increase in AI in tumours post-treatment may be a useful surrogate tumour marker of response to chemotherapy. For example, AI has been shown to correlate with pathological response in breast cancer biopsies collected after therapy [44], [45]. Some studies have suggested that immunohistochemistry for cleaved caspase-3 might replace measures of apoptosis such as TUNEL [46], [47], and absence or low numbers of cleaved caspase-3 positive cells have been associated with a worse prognosis in patients with nasopharyngeal carcinoma [48] and glioma [49]. However the transient expression of cleaved caspases and homology between the cleavage sites [50] may limit the clinical utility of IHC for cleaved caspases as predictive biomarkers. In contrast we have examined the hypothesis that expression of full-length caspases-8, -9 and -10 may be surrogate markers of tumour apoptotic potential, response to treatment and outcome. We have found considerable heterogeneity in the expression of these effectors of cell death, with no correlation between expression of the different caspases. Expression of caspase 8 and 10 was not significantly associated with OS or RFS. There was however a non-significant trend towards patients with tumours expressing low levels of caspase-9 protein having a worse RFS, which may reflect the importance of caspase-9 as an effector molecule of apoptotic cell death following treatment with current therapeutics. Low levels of caspase-9 protein in primary tumour could therefore identify patients that are most likely to benefit from non-caspase-9 dependent treatments, such as death receptor (DR)-mediated apoptosis through caspase-8 cleavage and induction of the non-mitochondrial apoptotic pathway [51], [52]. Consistent with this hypothesis ESFT are reported to be sensitive to TRAIL-induced apoptosis in preclinical models of ESFT growth [53]–[55]. Heterogeneous expression was not explained by differential methylation of the *CASP8* or *CASP9* gene promoters (Figure S2; [56]).

This study highlights the value of PI as a predictive biomarker of RFS and OS in patients with ESFT when measured in primary tumour. PI remained predictive of OS in a multivariate analysis, indicating that PI provides additional prognostic information independent of clinical markers used in current practice. This observation was authentic in a continuous statistical model, demonstrating that survival worsens as PI increases and therefore that risk can be defined most accurately based on PI as a continuous measure rather than choosing an arbitrary cut-point. The introduction of this assay into the pathological examination of ESFT at diagnosis may improve the ability to identify those patients destined to do badly that might benefit from alternative investigational treatment.

## Supporting Information

Figure S1 **Kaplan Meier survival plot to compare the relapse-free survival of patients with tumours that had low caspase-9 expression to that of patients with tumours that had high caspase-9 expression, p = 0.0538; log rank test.** Crosses identify censored events.
(TIF)

Figure S2 **Methylation of the CASP9 promoter in ESFT.** PCR products in lanes labelled U and M indicate the presence of unmethylated and methylated *CASP9* promoter regions respectively. DNA extracted from peripheral blood (PB) from healthy volunteers was included as an unmethylated control and CpG Methylase treated peripheral blood DNA (Meth PB) was used as a methylation control.
(PDF)

Table S1 **Comparison between frozen and FF-PE tumours and their distribution by age at diagnosis, tumour site and PI.**
(DOC)

Table S2 **Summary of patient and tumour information, PI, AI and protein expression.** Details of the 105 ESFT samples analysed with clinical information, PI and AI and expression of caspases-8, -9 and -10.
(XLS)
