# Supplementary figures and images for: Proliferation Index: A Continuous Model to Predict Prognosis in Patients with Tumours of the Ewing's Sarcoma Family
Source: PLoS One. 2014 Aug 26;9(8):e104106. doi: 10.1371/journal.pone.0104106 (PMC4144797; doi:10.1371/journal.pone.0104106)

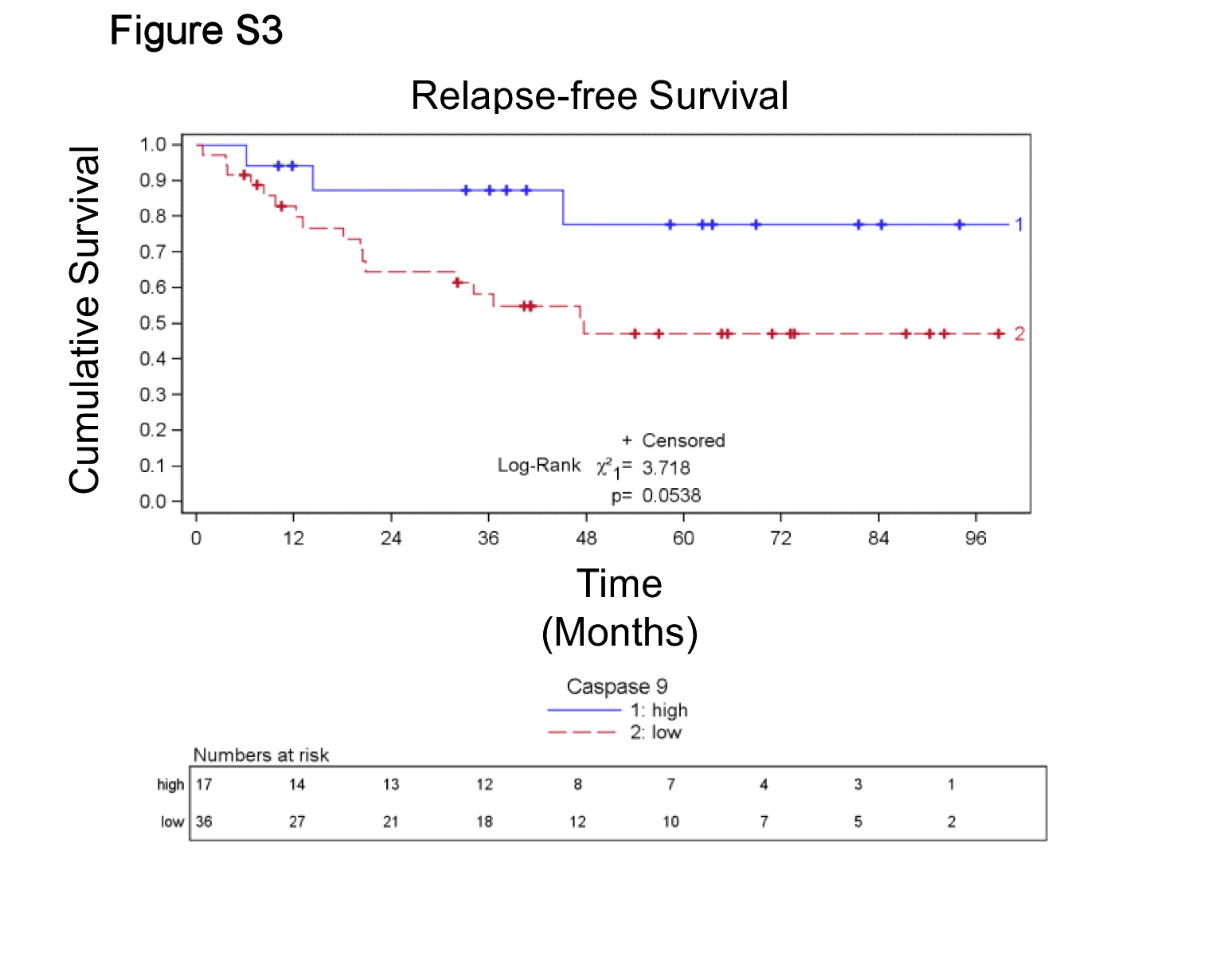

Supplement: Figure S1 — Kaplan Meier survival plot to compare the relapse-free survival of patients with tumours that had low caspase-9 expression to that of patients with tumours that had high caspase-9 expression, p = 0.0538; log rank test. Crosses identify censored events. (TIF) [file pone.0104106.s001.tif]

Figure S4

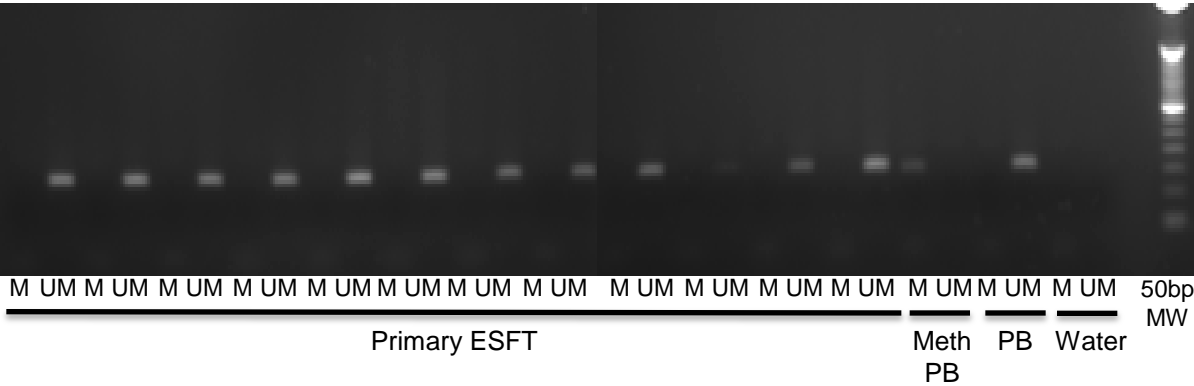

Supplement: Figure S2 — Methylation of the CASP9 promoter in ESFT. PCR products in lanes labelled U and M indicate the presence of unmethylated and methylated CASP9 promoter regions respectively. DNA extracted from peripheral blood (PB) from healthy volunteers was included as an unmethylated control and CpG Methylase treated peripheral blood DNA (Meth PB) was used as a methylation control. (PDF) [file pone.0104106.s002.pdf]
