# Supplementary material for: Proliferation Index: A Continuous Model to Predict Prognosis in Patients with Tumours of the Ewing's Sarcoma Family
Source: PLoS One. 2014 Aug 26;9(8):e104106. doi: 10.1371/journal.pone.0104106 (PMC4144797; doi:10.1371/journal.pone.0104106)
Supplement: Table S1 — Comparison between frozen and FF-PE tumours and their distribution by age at diagnosis, tumour site and PI. (DOC) [file pone.0104106.s003.doc]

**Table S1.**

| **Sample type** | **Age** | | **Tumour site** | | **Tumour volume** | | **PI** | |
| --- | --- | --- | --- | --- | --- | --- | --- | --- |
|  | **<14** | **≥14** | **Pelvic** | **Other** | **<200 ml** | **≥200 ml** | **<25** | **≥25** |
| **Frozen** | 51 (59.3%) | 35 (40.7%) | 21 (24.4%) | 62 (72.1%) | 28 (32.6%) | 41 (47.7%) | 58 (67.4%) | 24 (27.9%) |
| **FF-PE** | 11 (57.9%) | 8 (42.1%) | 2 (10.5%) | 11 (57.9%) | 9  (47.4%) | 5  (26.3%) | 12 (63.2%) | 6 (31.6%) |
| **Total** | 62 (59.0%) | 43 (41.0%) | 23 (21.9%) | 73 (69.5%) | 37 (35.2%) | 46 (43.8%) | 70 (66.7%) | 30 (28.6%) |
